# An Efficient Clustered Regularly Interspaced Short Palindromic Repeat (CRISPR)/CRISPR-Associated Protein 9 Mutagenesis System for Oil Palm (*Elaeis guineensis*)

**DOI:** 10.3389/fpls.2021.773656

**Published:** 2021-11-22

**Authors:** Wan-Chin Yeap, Norkhairunnisa Che Mohd Khan, Norfadzilah Jamalludin, Muhammad Rashdan Muad, David Ross Appleton, Harikrishna Kulaveerasingam

**Affiliations:** ^1^Sime Darby Plantation Technology Centre Sdn. Bhd., Serdang, Malaysia; ^2^Sime Darby Plantation Research Sdn. Bhd., Banting, Malaysia

**Keywords:** genome editing, CRISPR/Cas9, oil palm, *EgPDS*, *EgBRI1*

## Abstract

The clustered regularly interspaced short palindromic repeat (CRISPR)/CRISPR-associated protein 9 (Cas9) system has emerged as a powerful tool for the precise editing of plant genomes for crop improvement. Rapid *in vitro* methods for the determination of guide RNA (gRNA) cleavage efficiency and an efficient DNA delivery system is essential for gene editing. However, we lack an efficient gene-editing system for palm species. In this study, we described the development of a transient oil palm protoplast assay to rapidly evaluate the cleavage efficiency of CRISPR/Cas9 mutagenesis and the generation of stable transformed oil palms using biolistic particle bombardment in immature embryos. Using the *phytoene desaturase* (*EgPDS*) gene, we found cleavage frequency of up to 25.49% in electro-transfected protoplast, which enables the production of transgenic oil palm shoots exhibiting chimeric albino phenotypes as a result of DNA insertions, deletions (InDels), and nucleotide substitutions, with a mutation efficiency of 62.5–83.33%. We further validated the mutagenesis efficiency and specificity of the CRISPR/Cas9 system in oil palm by targeting the *brassinosteroid-insensitive 1* (*EgBRI1*) gene, which resulted in nucleotide substitutions in *EgBRI1* with premature necrosis phenotype in oil palm transgenic shoots and stunted phenotype resulting from DNA InDels. Taken together, our results showed that effective and efficient editing of genes using the CRISPR/Cas9 system can be achieved in oil palm by optimizing the selection of efficient gRNA and DNA delivery methods. This newly designed strategy will enable new routes for the genetic improvement in oil palm and related species.

## Introduction

Genome editing technology is emerging as a powerful tool to introduce accurately targeted mutations for plant gene function studies and provide new avenues for crop improvement due to its simplicity, flexibility, consistency, accuracy, and high efficiency (Lozano-Juste and Cutler, 2014; Voytas and Gao, 2014; Bortesi and Fischer, 2015). It has been made simpler with clustered regularly interspaced short palindromic repeat (CRISPR)/CRISPR-associated protein 9 (Cas9) system due to its versatility, effectiveness, and efficiency (Feng et al., 2013; Ma et al., 2015; Jiang and Doudna, 2017). The CRISPR/Cas9 system has been successfully established and widely applied in various economically important crops, including wheat, rice, maize, brassica, sweet potato, banana, and grapevine, to enhance crop productivity, grain quality, nutritional value, disease tolerance, resilience to climate change, and herbicide resistance (Shi et al., 2017; Macovei et al., 2018; Okuzaki et al., 2018; Zhang et al., 2018; Wang et al., 2019; Kaur et al., 2020; Li et al., 2020).

Clustered regularly interspaced short palindromic repeat/Cas9 mediates precise genome modification by gene knock in, knock out, base editing, transcriptional activation or repression, epigenetic modification, and RNA editing (Feng et al., 2013; Konermann et al., 2015; Zong et al., 2017; Mao et al., 2018; Papikian et al., 2019). The CRISPR/Cas9 endonuclease protein is directed to a specific chromosomal DNA site by a short sequence-specific single guide RNA (gRNA) to induce DNA double-stranded breaks (DSBs) and enable precise editing of the target DNA sequence with mutation events, including insertions, deletions (InDels), and nucleotide substitutions induced by the cell repair mechanisms, non-homologous end-joining (NHEJ), or homology-directed repair (HDR) (Jiang and Doudna, 2017). In plants, the efficiency of CRISPR/Cas9-mediated mutagenesis is affected by the editing components (Cas9, promoter, gRNA design, and specificity), the transformation or delivery method into plant cells, the ability of plants to regenerate, and the sensitivity of the mutation detection method (Ma et al., 2015; Liu et al., 2016; Thyme et al., 2016; Wang et al., 2018; Montecillo et al., 2020). The CRISPR/Cas9 system can be delivered into plant cells using several methods, including plant transformation of CRISPR/Cas9 and gRNA expression cassettes, virus-mediated gRNA delivery, DNA-free CRISPR–Cas9 ribonucleoprotein (RNP) delivery to protoplasts, and *de novo* meristem induction bypassing tissue culture process (Xu et al., 2014; Ali et al., 2015; Woo et al., 2015; Wang et al., 2018; Montecillo et al., 2020). The efficiency of CRISPR/Cas9 is variable in different plant species and requires fine-tuning of the conditions to achieve successful and highly efficient genome editing.

Global food demand is expected to increase by 60% in 2050, driven by the rapid expansion of the human population reaching approximately 9–11 billion and global dietary shifts intensifying wellness and value addition (Fróna et al., 2019). Oil palm is well recognized as the most efficient oil crop that contributes to 35% of global vegetable oil; thus, it can offer a critical solution for global food insecurity to feed the growing global population (Mielke, 2017). Despite the yield and oil quality improvement achieved through conventional breeding and marker-assisted breeding in the past decades (Soh et al., 2017), the annual growth in palm oil production is likely to slow down in the coming years due to a deceleration in land expansion, yield stagnation, climate change, declining labor force, increase in production costs, and pest and disease issues. Thus, the application of genome editing technology can provide opportunities to accelerate and enhance breeding programs with high precision in oil palm to achieve food security and sustainable agriculture.

Limitations in an efficient genetic transformation system, a plant regeneration process, and an *in vitro* testing system hindered the establishment of the CRISPR/Cas9 system in oil palm. Here, we report an establishment of the CRISPR/Cas9 system that enables efficient genome editing in oil palm. We investigated the efficacy and efficiency of a codon-optimized CRISPR/Cas9 by targeting oil palm *phytoene desaturase* (*EgPDS*) gene as a phenotypic marker to facilitate rapid screening of mutant lines as mutation of the *PDS* gene disrupts the carotenoid pathway and results in albino and dwarf phenotypes (Odipio et al., 2017). We first established a rapid *in vitro* detection method for testing gRNA efficiency in oil palm using protoplast electro-transfection. Subsequently, we demonstrated the delivery of CRISPR/Cas9 and gRNA cassettes into oil palm immature embryos using biolistic particle bombardment-mediated transformation and validated the mutagenesis effects of CRISPR/Cas9, both genotype and phenotype in regenerated oil palms. We further validated the mutagenesis efficiency and specificity of the CRISPR/Cas9 system in oil palm by targeting oil palm *brassinosteroid-insensitive 1* (*EgBRI1*). We showed that mutations in the kinase domain of BRI1 are associated with palms displaying stunted phenotype and necrosis at the leaf apex.

## Materials and Methods

### Plant Materials

Oil palm *dura* (Deli *dura*) immature embryos extracted from the fresh oil palm fruits at 12 weeks after pollination (WAP) were supplied by Oil Palm Breeding, Sime Darby Plantation Research Sdn. Bhd., Banting, Malaysia, and used as the starting material for this study. Before particle bombardment with plasmids at 200 replicates/constructs, the immature embryos were cultured on Murashige and Skoog (1962) media with sucrose and vitamins. Unopened oil palm spear leaf was used for protoplast isolation.

### Construction of Clustered Regularly Interspaced Short Palindromic Repeat/CRISPR-Associated Protein 9 and Guide RNA Vector

**FIGURE 1:**
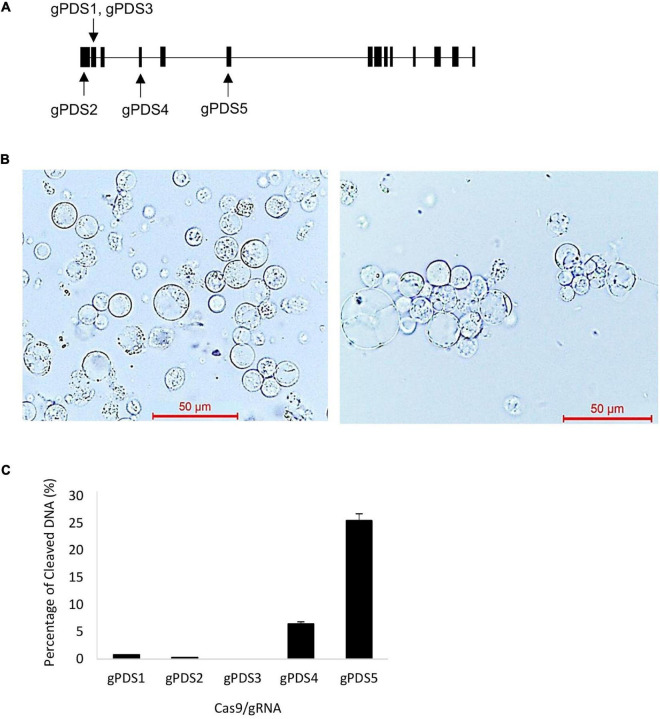
Efficiency testing of clustered regularly interspaced short palindromic repeat (CRISPR)/CRISPR-associated protein 9 (Cas9) and guide RNA (gRNA) activity using electro-transfection in oil palm protoplasts. **(A)** Schematic map of gRNA target site in *phytoene desaturase* (*EgPDS)* gene. **(B)** Oil palm protoplast before (left) and protoplast agglutination after (right) electro-transfection with Cas9/gRNA. **(C)** Cleavage efficiency of Cas9/gRNA in oil palm protoplast following electro-transfection. Data are average values ± SE from four independent experiments.

The *Streptococcus pyogenes Cas9* nuclease gene (Jinek et al., 2012) was codon optimized (Supplementary Data Sheet 1) by replacing the tandem rare codons in native gene to codon usage bias in oil palm using the OptimumGene™ codon optimization analysis (GenScript USA Inc., Nanjing, China) to enhance the efficiencies in *Cas9* gene expression and translation in oil palm. The codon-optimized *Cas9* was synthesized together with the Gateway attP1 site and nuclear localization signal (NLS) fused to the 5′ of *SpCas9* and Gateway attP2 site fused to the 3′ of the gene (GenScript USA Inc., Nanjing, China). The synthesized gene construct was shuttled into the pXHb7SNFI-UBIL vector (Vlaams Instituut voor Biotechnologie, Ghent, Belgium) fused to the *Ubiquitin* promoter and β*-glucuronidase* (*GUS*) reporter using Gateway^®^ BP Clonase™ II enzyme mix (Thermo Fisher Scientific, Waltham, MA, United States). The resulting construct is known as pUbil-Cas9 (Supplementary Figure 1). The potential target sequences of gRNAs for oil palms *EgPDS* (accession no. XP_010937221) and *EgBRI1* (accession no. XP_010927763) were analyzed and designed using Benchling^1^ and CCTop^2^ (Stemmer et al., 2015). These gRNAs were selected based on their position in the gene, the predicted efficacy score, and the potential for off-target mutation. These gRNAs (Supplementary Table 1) designed to target different exon regions of the *EgPDS* gene (Figure 1A) were cloned into a modified pCAMBIA1201 vector, fused to rice *OsU3* promoter and gRNA scaffold cassette (Supplementary Figure 1).

### Protoplast Electroporation Transformation and Detection of Guide RNA Efficiency

The oil palm mesophyll protoplasts were isolated from the unopened spear leaf as described previously (Masani et al., 2013). These protoplasts were co-transformed with 2 μg plasmids of pUbil-Cas9 and individual gRNA constructed for *EgPDS* and *EgBRI1* genes using electroporation. A single vector of pUbil-Cas9 or gRNA was transformed as controls. The protoplast and plasmid mixture in HEPES-buffered saline solution was electroporated using Gene Pulser Xcell electroporation system (Bio-Rad Laboratories, Hercules, CA, United States) with a square wave pulse program: 550 V/cm (220 V setting/0.4 cm width), 10 ms for two times, and a 20 ms interval for poring pulse. The electroporated protoplasts were incubated in HEPES-buffered saline solution under dark conditions at 25°C with gentle swirling for 72 h. The protoplasts were washed using a washing solution, and the genomic DNA of transformed protoplasts were extracted using QuickExtract™ DNA Extraction Solution (Lucigen, Middleton, WI, United States) according to the instructions of the manufacturer. The electro-transfection efficiency was determined using absolute quantitative real-time PCR. The DNA copies of the *GUS* reporter gene transformed into protoplast were compared against the total DNA copies of the endogenous gene, *Cyclophilin 2* in protoplast, both DNA copies were determined from plasmid dilution standard curve. According to the instructions of the manufacturer, the target site region, flanking the gRNAs of *EgPDS* and *EgBRI1* genes, was amplified, respectively, from the transformed and wild-type protoplasts DNA using region-specific primers (Supplementary Table 2) and iProof™ High-Fidelity DNA Polymerase (Bio-Rad Laboratories, Hercules, CA, United States). The mutations were detected by comparing amplified fragments from the transformed and wild-type protoplasts (50:50 ratio) using the AccuCleave™ T7CE Kit and detection using CRISPR Discovery Gel Kit and Fragment Analyzer™ Automated CE System (Agilent Technologies, Palo Alto, CA, United States). The mutation frequency of the protoplast population was determined based on the percentage of cleavage efficiency calculated using the PROsize Data Analysis Software (Agilent Technologies, Palo Alto, CA, United States).

### Biolistic Transformation and Plant Regeneration

The pUbil-Cas9 and two efficient gRNA constructs for *EgPDS* and *EgBRI1* (2 μg each) were coated on the surface of 3 mg gold particles in 50% glycerol and mixed with 1M CaCl_2_ and 16 mM spermidine. The mixture was washed with absolute ethanol three times and was resuspended in 60 μl absolute ethanol. For each bombardment, 10 μl of DNA-microcarrier mixture was placed on the microcarrier and bombarded two times into oil palm immature embryos using the PDS-1000/Hepta Biolistic Particle Delivery System (Bio-Rad Laboratories, Hercules, CA, United States) with 1,100 psi rupture disks, vacuum at 27 mmHg pressure, and 9 cm distance from the tissue. Individual pUbil-Cas9, gRNA, and the combinations of both constructs were bombarded or co-bombarded into 200 replicates of immature embryos. The pUbil-Cas9 and individual gRNAs were used as controls. The transformed immature embryos were maintained in MS agar with sucrose and vitamins (Sigma-Aldrich, St. Louis, MO, United States) supplemented with 50 μg/ml of hygromycin B. Regenerated plants were transferred into MS agar without antibiotic or herbicide and cultured under 16-h light/8-h dark photoperiod for 6–8 months.

### Identification of Transgene and Detection of Clustered Regularly Interspaced Short Palindromic Repeat/CRISPR-Associated Protein 9 Mutations

Transgenic plants were identified by their resistance to hygromycin at the early phase of tissue culture. Three transformed embryos were randomly selected from the controls and confirmed using β-glucuronidase staining after 2 months on selection media. Putative transgenic plants were confirmed using the Phire Plant Direct PCR Kit (Thermo Fisher Scientific, Waltham, MA, United States) and specific primers targeting *Cas9* and *OsU3* promoter sequences (Supplementary Table 3). Five transgenic oil palm mutants with altered phenotypes were randomly selected for sequence analysis. Their genomic DNA was extracted using QuickExtract™ DNA Extraction Solution (Lucigen, Middleton, WI, United States). The target site of *EgPDS* and *EgBRI1* was amplified using region-specific primers (Supplementary Table 2) and iProof™ High-Fidelity DNA Polymerase (Bio-Rad Laboratories, Hercules, CA, United States). Amplicons were purified and analyzed using Sanger sequencing (Applied Biosystems, Foster City, CA, United States) for the detection of specific InDels and substitution. Mutation efficiency for each gRNA was evaluated based on the number of T0 transgenic plants with mutation compared to the number of genotyped T0 transgenic plants. The mutation event and frequency were analyzed and detected using TIDE^3^ (Brinkman et al., 2014). These amplified fragments were cloned into pJET1.2 (Thermo Fisher Scientific, Waltham, MA, United States), and 20 colonies were randomly selected for single-colony plasmid sequencing for chimeric mutation assessment. The sequences were analyzed by aligning to wild-type sequences as reference.

## Results

### Development of a Highly Efficient System for Genome Editing in Oil Palm

To establish a highly efficient CRISPR/Cas9 system in oil palm, we targeted *EgPDS* as genetic lesions will result in visible phenotypes. Five gRNAs were designed to target mutagenesis at different exon regions of the *EgPDS* gene (Figure 1A). First, we examined the functionality of codon-optimized SpCas9 and the effectiveness of the five gRNAs to identify efficient gRNA for further phenotypic validation in the transgenic oil palm, using a fast electroporation-mediated protoplast transient expression system. The protoplast electro-transfection efficiencies ranged from 17 to 26%. Agglutination among protoplasts was observed after electroporation with target constructs without damages (Figure 1B). In *EgPDS* mutagenesis, mutations were detected in protoplasts transformed with Cas9/gPDS4 and Cas9/gPDS5 with cleavage frequencies of 6.49 ± 0.7 and 25.49 ± 4.11%, respectively (Figure 1C). In contrast, we found minimal mutagenesis activity for gPDS1, gPDS2, and gPDS3 gRNAs, and no mutation activity was detected in the control protoplast pool. These results suggest that the codon-optimized Cas9 and gRNAs are an efficient system for mutagenesis in oil palm and that their efficiency can be easily determined by protoplast electro-transfection.

**FIGURE 2:**
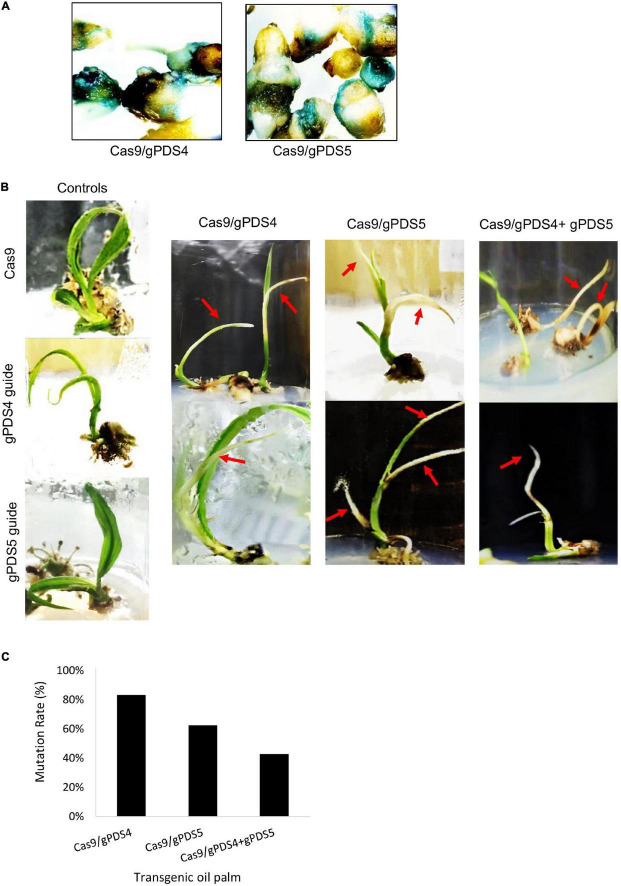
Mutagenesis mediated by CRISPR/Cas9 and gRNA of *EgPDS* in *in vitro* transgenic oil palm. **(A)** Confirmation of genetic transformation by β*-glucuronidase* (GUS) assay in immature embryos transformed with Cas9/gPDS4 and Cas9/gPDS5. **(B)** Phenotypes of the Cas9/gPDS4 or gPDS5-mediated mutagenesis and control transgenic *in vitro* oil palms. Arrow indicating shoots with albino phenotypes. **(C)** Mutation frequencies of CRISPR/Cas9 mutagenesis in *in vitro* transgenic oil palms.

To examine the efficiency of CRISPR/Cas9 in oil palm, we generated stable transgenic plants by the transformation of pUbil-Cas9 and highly efficient gRNAs for *EgPDS* (gPDS4 and gPDS5) using the biolistic particle bombardment method. Putatively transformed embryos were selected from non-transformants based on their survival on a hygromycin selection medium. A total of 14 putative transformants were generated using pUbil-Cas9 and gPDS4 transformation, whereas 13 putative transformants were generated using pUil-Cas9 and gPDS5 transformation, with a transformation efficiency of 7 and 6.5%, respectively (Table 1). The transformation of a single vector cassette for Cas9 or individual gRNA into immature embryos resulted in a relatively higher transformation efficiency of up to 19.5%. We randomly selected the putative transformed immature embryos for GUS histochemical assay (Figure 2A). The hygromycin-resistant embryos were later transferred to negative selection media to induce shoot regeneration. Notably, we found that the regeneration efficiencies for *EgPDS-*edited plants ranged from 44 to 77%.

**TABLE 1:** The efficiency of clustered regularly interspaced short palindromic repeat (CRISPR)/CRISPR-associated protein 9 (Cas9) and guide RNA (gRNA) transformation targeting *phytoene desaturase* (*EgPDS)* gene in oil palm and the phenotypes of the transgenic plants.

Gene	Guide RNA	Bombarded immature embryo	Putative transformant	Regenerated T0 shoots	T0 with mutation/genotyped T0 plants	Mutation rate	Transformants with altered phenotype
Control	*Cas9*	200	22 (11%)	17 (77%)	0/5	0	0
	*gPDS4*	200	39 (19.5%)	29 (74%)	0/5	0	0
	*gPDS5*	200	15 (7.5%)	10 (67%)	0/5	0	0
*EgPDS*	*Cas9/gPDS4*	200	14 (7%)	9 (64%)	5/6	83.33%	8 chimeric albino (89%)
	*Cas9/gPDS5*	200	13 (6.5%)	10 (77%)	5/8	62.50%	7 chimeric albino (70%)
	*Cas9/gPDS4* + *gPDS5*	200	18 (9%)	8 (44%)	3/7	42.86%	3 chimeric albino (37.5%)

**FIGURE 3:**
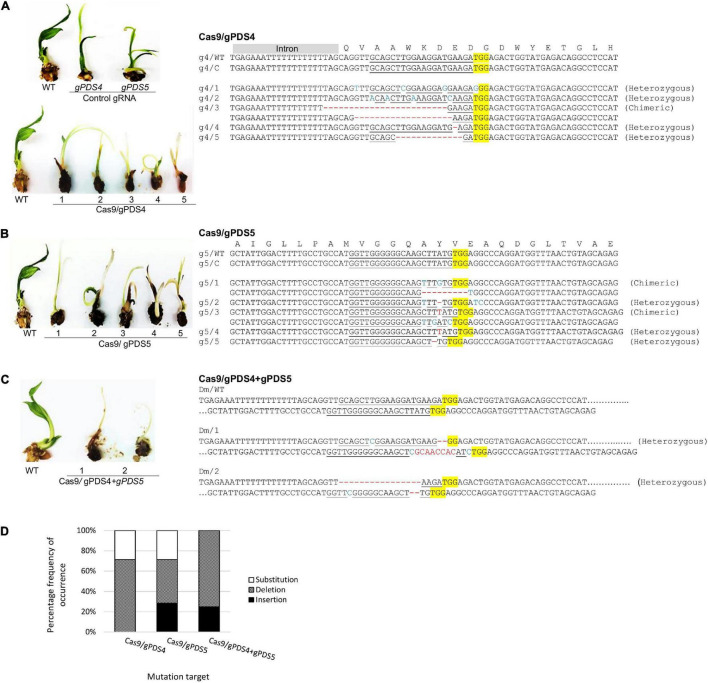
Mutation events of targeted CRISPR/Cas9 mutagenesis of *EgPDS* gene in *in vitro* transgenic oil palm. Mutation events detected in the transgenic oil palms exhibiting the chimeric albino phenotypes using sequencing-based detection at the target sites of **(A)** gPDS4, **(B)** gPDS5, and **(C)** both gPDS4 and gPDS5 in the *EgPDS* gene and **(D)** the percentage frequency of occurrence for each type of mutation event. Protospacer adjacent motif sequence (PAM) is highlighted in yellow, InDels sequences of the target site are shown in red, and nucleotide substitution are marked in blue. g4, gPDS4; WT, wild-type; C, control; g5, gPDS5; Dm, double mutant; 1–5, plantlet number.

Alteration of *PDS* gene function is predicted to result in albino phenotypes due to defects in carotenoid biosynthesis (Mann et al., 1994). We found that the shoots of regenerated oil palms transformed with pUbil-Cas9/gPDS4 and pUbil-Cas9/gPDS5 displayed albino sectors indicating mosaicism (Figure 2B). These transgenic plantlets were scored based on their albino phenotype to provide a preliminary mutation efficiency for each gRNA module. Mutagenesis mediated by pUbil-Cas9/gPDS4 resulted in 83.3% mutation with chimeric albinism phenotype (Figure 2C) observed in 8 out of 9 regenerated oil palm shoots (Table 1). A total of 7 out of 10 regenerated oil palm shoots exhibited a similar chimeric albinism phenotype with Cas9/gPDS5 mutagenesis. Control plantlets transformed with a single construct of pUbil-Cas9/gRNA did not display signs of albinism. To investigate the effect of multiple mutageneses on plant regeneration and phenotype, we generated mutants transformed with pUbil-Cas9 and both gRNA (gPDS4 and gPDS5) constructs. Notably, only eight hygromycin-resistant immature embryos with gRNA double mutations regenerated into shoots. The EgPDS double mutants showed delayed shoot regeneration compared with the single gRNA mutants and non-edited control equivalents, nevertheless, the albino phenotype was noticeable in the shoots of the three mutants (Table 1 and Figure 2B). All regenerated shoots with albino phenotypes were tested positive for the presence of both Cas9 and gRNA using PCR analysis. To confirm the gene-editing events, we performed PCR amplification and Sanger sequencing analysis using shoots exhibiting a clear EgPDS-edited albino phenotype (Figures 3A–C). These sequence traces were first analyzed using the TIDE algorithm (Brinkman et al., 2014) to identify the occurrence of InDels from the DSBs (Supplementary Figure 2). The sequence data indicated multiple mutations including InDels and single base substitutions in the EgPDS gene of the genotyped regenerated shoots, and the mutated sequences were validated using gene cloning. Mutations by Cas9/gPDS4 induced independent events with 71% occurrence of deletions (Figure 3D) ranging from −1 to −24 bp and 29% of nucleotide substitutions upstream of the protospacer adjacent motif sequence (PAM) region of the target site. The largest deletion (−24 bp) was detected at the boundary of intron 3, and exon 4 of the EgPDS gene may cause disruption in the splice-regulatory sequences and modulate exon skipping or alter splicing of pre-mRNA. Insertion (+1 bp), deletion (−1, −2, and −9 bp), and substitution (1–3 bp) were detected in Cas9/gPDS5 mutants at an occurrence frequency of 29, 43, and 29%, respectively. The nucleotide indels led to disruption in amino acid sequence and exon skipping in one of the mutants, whereas amino acid substitutions resulted from the nucleotide substitutions. In double mutation shoots, deletion (−2 and −16 bp) (75% occurrence) and insertion (+8 bp) (25% occurrence) were detected at the gPDS4 and gPDS5 target regions. These mutations led to amino acid frameshifts and the introduction of multiple stop codons near target regions. Collectively, our data suggest that the optimization of vectors for efficient Cas9 and gRNA activity combined with a rapid transformation system is a critical factor for an efficient gene-editing system in oil palm.

### Targeted Mutagenesis of *Brassinosteroid-Insensitive 1* to Manipulate Brassinosteroid Responses in Oil Palm

**FIGURE 4:**
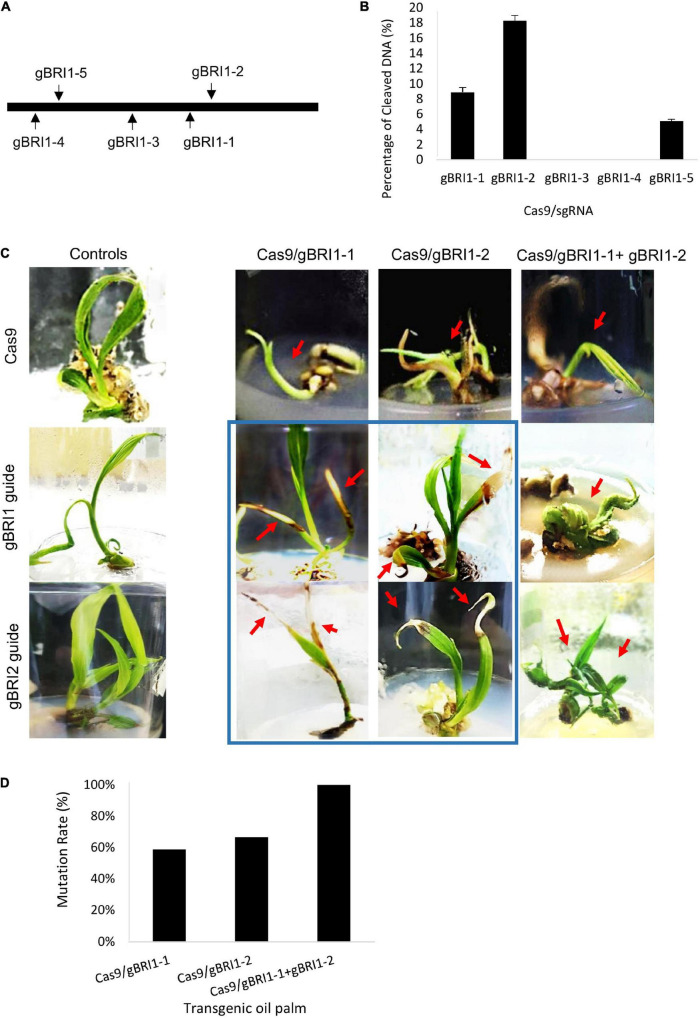
Clustered regularly interspaced short palindromic repeat/Cas9-targeted mutagenesis of *brassinosteroid-insensitive 1(EgBRI1)* gene in *in vitro* oil palm plantlets. **(A)** Schema of gRNA targeting intronless *EgBRI1* gene in oil palm. **(B)** Cleavage efficiency of Cas9/gRNA targeting *EgBRI1* gene in oil palm protoplast. Data are average values ± SE from four independent experiments. **(C)** CRISPR/Cas9-induced *EgBRI1* mutations at different gRNA target sites showing stunted growth and leaf apex necrosis phenotypes (boxed in blue) in transgenic oil palm shootlets and controls transformed with either Cas9 or gRNA alone. **(D)** Mutation frequencies of CRISPR/Cas9 mutagenesis based on the occurrence of stunted or necrotic leaf apex phenotypes in transgenic oil palm.

**FIGURE 5:**
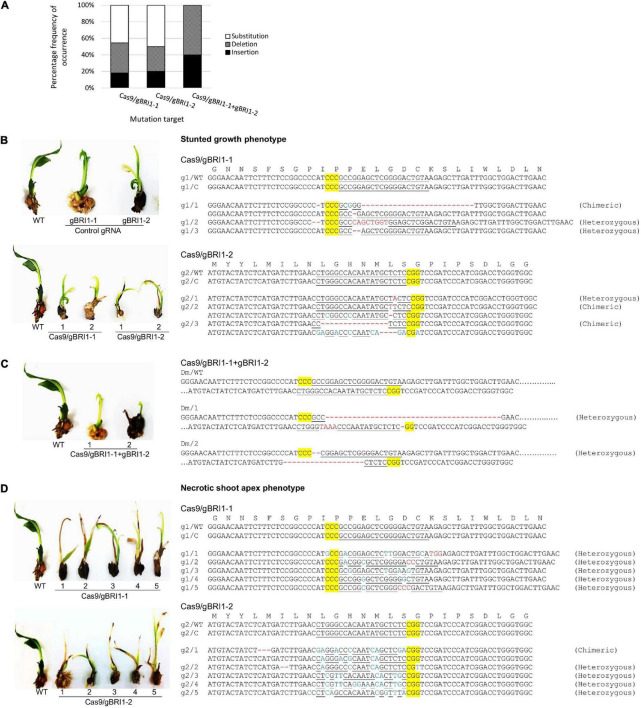
Clustered regularly interspaced short palindromic repeat/Cas9-mediated mutation events and phenotypes of *EgBRI1* mutant transgenic oil palm. **(A)** The overall percentage frequency of occurrence for each type of mutation event detected at gBRI1-1 and gBRI1-2 targeted sites. The mutation events detected in the transgenic oil palms exhibiting **(B)** stunted growth phenotype induced by CRISPR/Cas9 and gBRI1-1, gBRI1-2, or **(C)** dual gRNAs mutation induction in the *EgBRI1* gene, and **(D)** necrotic shoot apex phenotype. PAM is highlighted in yellow, InDels sequences of the target site are shown in red, and nucleotide substitution is marked in blue. g1, gBRI1-1; g2, gBRI1-2; WT, wild-type; C, control; Dm, double mutant; 1–5, plantlet number.

*BRI1* gene encodes a brassinosteroid receptor and defective in *BRI1* results in dwarf phenotype in *Arabidopsis*, rice, and maize (Clouse et al., 1996; Noguchi et al., 1999; Yamamuro et al., 2000; Kir et al., 2015). Hence, we selected dwarfism as another visual phenotypic marker for the validation of the genome editing system in oil palm. To manipulate the brassinosteroid responses, we selected *EgBRI1* (accession no. XP_010927763) for targeted mutagenesis. The *EgBRI1* genomic DNA contains one intronless open reading frame of 3390 bp, and gRNAs were designed based on PAM sites (Figure 4A). The activity of the CRISPR/Cas9 system and the efficiencies of gRNAs targeting the *EgBRI1* gene were first verified using *in vitro* electro-transfection system in protoplast. Mutagenesis of *EgBRI1* gene mediated by Cas9 with gBRI1-1, gBRI1-2, and gBRI1-5 showed cleavage frequencies of 9.8 ± 1.21, 18.32 ± 1.4, and 5.1 ± 0.53%, respectively, in protoplast with electro-transfection efficiencies ranging from 12 to 17% (Figure 4B). However, cleavage was not detectable with the remaining gRNAs tested in the protoplast system. The two most efficient gRNAs, gBRI1-1 and gBRI1-2, were selected for transformation in the oil palm. The transformation with pUbil-Cas9 and gBRI1-1 resulted in 24 putative transformants (with a 12% transformation efficiency), while the transformation with pUbil-Cas9 and gBRI1-2 resulted in 23 putative transformants (with an 11.5% transformation efficiency) (Table 2). Overall, the mutagenesis efficiency for *EgBRI1* ranged between 58.82 and 100%. Targeted mutagenesis of the *EgBRI1* resulted in two distinct phenotypes found in most of the regenerated plants (75–87% regeneration efficiency), reduced plant elongation (stunted), and apex necrosis (Figures 4C,D) with mutation efficiency of 58.82 and 66.67% using pUbil-Cas9/gBRI1-1 and pUbil-Cas9/gBRI1-2, respectively. Sequence analysis of the targeted region detected a wide range of mutations in regenerated transgenic oil palm shoots. Those that arose from pUbil-Cas9/gBRI1-1 and pUbil-Cas9/gBRI1-2 had multiple nucleotide insertions (+1, +8 bp), nucleotide deletions (−1 to −25 bp), and nucleotide substitutions at the sequence targeted at an occurrence frequency of 18–20, 30–36, and 45–50%, respectively (Figures 5A,B). These nonsense mutations may cause the frameshift and aberrant amino acid sequences with premature stop codon introduction and lead to protein truncation. Likewise, the impaired growth phenotypes in mutants generated using paired-gRNAs resulted primarily in deletions (−1, −2, −18, and −39 bp) and insertions (+4 bp) (Figure 5C). Nucleotide substitution mutations are predicted to affect the activity of the leucine-rich repeat receptor-like protein kinase domain of BRI1 (Figure 5D) and are responsible for the apex necrosis phenotype observed in these plants.

**TABLE 2:** The efficiency of CRISPR/Cas9 and gRNA transformation targeting *brassinosteroid-insensitive 1 (EgBRI1)* gene in oil palm and the phenotypes of the transgenic plants.

Gene	Guide RNA	Bombarded immature embryo	Putative transformant	Regenerated T0 shoots	T0 with mutation/genotyped T0 plants	Mutation rate	Transformants with altered phenotype
Control	*Cas9*	200	22 (11%)	17 (77%)	0/5	0	0
	*gBRI1-1*	200	17 (8.5%)	13 (76.5%)	0/5	0	0
	*gBRI1-2*	200	10 (5%)	8 (80%)	0/5	0	0
*EgBRI1*	*Cas9/gBRI1-1*	200	24 (12%)	18 (75%)	10/17	58.82%	6 stunted (33%) 5 leaf necrosis (28%)
	*Cas9/gBRI1-2*	200	23 (11.5%)	20 (87%)	10/15	66.67%	5 stunted (25%) 10 leaf necrosis (50%)
	*Cas9/gBRI1-1* + *gBRI1-2*	200	36 (18%)	30 (83%)	7/7	100%	30 stunted (100%)

## Discussion

Genome editing provides opportunities for the improvement of the trait in crops, yet its implementation requires an efficient genetic transformation and regeneration system. In oil palm, limitations in the DNA delivery method, inefficient clonal propagation, and regeneration processes constrain rapid progress in genetic engineering. In this study, we developed an efficient system for CRISPR/Cas9 genome editing in oil palm by targeting *EgPDS* and *EgBRI1* genes. Mutation rates mediated by CRISPR/Cas9 varied depending on the specificity and effectiveness of gRNAs to target sequences, delivery methods, and sensitivity of the detection method. We first established an *in vitro* protoplast electro-transfection method to identify mutagenic and efficient gRNAs. Electroporation of Cas9 and gRNA constructs using our optimized conditions resulted in cleavage frequencies of up to 25.49% in viable oil palm protoplasts, suggesting that the codon-optimized Cas9 was effective in inducing cleavage. Several gRNAs failed to elicit cleavage despite high GC-content has been associated with higher Cas9 editing efficiency (Liu et al., 2016). This may be contributed by the sequence composition or secondary structure of several target gRNAs that potentially cause failure to recognize target sequence or formation of functional Cas9-gRNA complex in protoplast (Thyme et al., 2016). In cabbage, neon electro-transfection at 1,000 V was 1.4 times more efficient than PEG-mediated transfection in delivering CRISPR-Cas9 RNP into protoplast and resulted in 3.4% InDels mutation frequency in the *PDS1* gene (Lee et al., 2020). Despite a previously reported PEG-mediated transfection method for protoplast, the protoplast viability and reproducibility are significantly reduced due to toxicity effects of PEG (Masani et al., 2014). Our method for electro-transfection of genome editing tools in oil palm protoplasts provides an effective alternative strategy for gRNA cleavage activity and potential opportunity to develop DNA-free genome-edited palms. However, limitations with oil palm protoplast regeneration require further studies for improvement to enable deployment of the protoplast system for DNA-free gene editing.

High transformation efficiency gene editing has been reported in monocot plant species including 57% in maize, 80% in wheat, 64% in rice, and 14.3% in sorghum (Xu et al., 2014; Char et al., 2017, 2020; Macovei et al., 2018; Zhang et al., 2018). Previous studies in oil palm achieved 0.7–1.5% transformation efficiencies using *Agrobacterium-*mediated and microprojectile bombardment transformation methods in embryogenic callus and regeneration by indirect somatic embryogenesis (Parveez et al., 1998; Masli et al., 2012; Dayang Izawati et al., 2015). In this study, we obtained 5–19.5% transformation efficiencies using particle bombardment into zygotic embryos, and up to 87% of embryos regenerated into shoots through direct embryogenesis. Our results suggest that the optimization of transformation protocol, selection of explant, and regeneration methods are effective in improving oil palm transformation efficiency. Furthermore, a direct embryogenesis approach was employed to enable rapid regeneration with minimized risk of culture-induced genetic variation that commonly occurs during callusing phase (Weckx et al., 2019). Although chimeric albinism phenotype was observed in the transgenic *EgPDS*-edited palms using our method, nevertheless, homozygous mutants and transgene removal can be obtained by genetic segregation and backcrossing.

We achieved high editing efficiency (42.86–100%) at target sites of *EgPDS* and *EgBRI1* genes in transgenic oil palms with our CRISPR/Cas9 system. In rice, high mutagenesis efficiency of 87–100% (di-allelic edits) was achieved using rice codon-optimized SpCas9 compared with the optimized SpCas9 from bacterial, human, and *Chlamydomonas* in T0 transgenic plants (Zhou et al., 2014). High editing efficiency in our system may be attributed to the oil palm codon-optimized *Cas9* gene driven by ubiquitin, an active promoter in monocot (Schledzewski and Mendel, 1994), efficient gRNAs, and delivery method for Cas9/gRNA. Our transgenic plants showed insertions (18–40%), deletions (30–75%), and high levels of nucleotide substitutions (29–50%) in *EgPDS* and *EgBRI1* genes. Although InDels are the most common mutation induced by error-prone NHEJ repair mechanism that involves direct rejoining of DSB ends regardless of sequence homology (Rodgers and McVey, 2016), high frequency of NHEJ-induced base substitutions are also prevalent in plants and substantial evidence have been reported in rice, cassava, citrus, soybean, and melon (Sun et al., 2015; Odipio et al., 2017; Macovei et al., 2018; Hooghvorst et al., 2019; Dutt et al., 2020). For instance, a high frequency of base substitutions has been reported in melon (91%) and rice (25–45%) (Macovei et al., 2018; Hooghvorst et al., 2019). In our study, InDels in the *EgBRI1* gene resulted in reduced plant elongation phenotype, while leaf apex necrosis was generated from base substitution events in transgenic palms. Early studies in *Arabidopsis* and rice reported dwarf and shortened internode phenotypes in the *bri1* loss-of-function mutant plants (Clouse et al., 1996; Noguchi et al., 1999; Yamamuro et al., 2000). Choe et al. (1999) further reported that mutation in *Arabidopsis bri1* results in a retarded leaf senescence phenotype. Phenotypes of our *EgBRI1* transgenic mutant palms are substantially affected by the types of NHEJ-induced mutations, and the specificity of the repair mechanism for DSBs is critical to driving accurate genomic changes without errors. Interestingly, we observed a low frequency of nucleotide substitutions using pUbil-Cas9/paired-gRNAs in both genes. Blunt end cleavage site generated using single gRNA is commonly repaired by error-prone NHEJ mechanism, but precise end-joining repairs have been reported in human cells through simultaneous DNA DSBs by Cas9/paired gRNAs, suggesting a direct ligation without end processing (Zheng et al., 2014). Moreover, previous studies in mammalian cells reported that the accuracy of the NHEJ repair mechanism is reduced when the distance between two collinear DSBs is beyond 1 kb (Boubakour-Azzouz and Ricchetti, 2008; Guirouilh-Barbat et al., 2016; Guo et al., 2018). Simultaneous DSBs by Cas9/paired-gRNAs at short distance may improve the specificity of the repair mechanism; however, the current understanding of this precise mechanism remains to be determined. Hence, the dynamic interaction between Cas9 cleavage and the endogenous DNA repair mechanism needs further study to enhance the efficiency and specificity of CRISPR/Cas9 system applications in plants.

Furthermore, we adapted the paired-gRNA using multi-cassette (monocistronic) transformation to increase the mutation frequency and chromosomal fragment deletion in the targeted gene. The assayed pUbil-Cas9/paired-gRNAs were mutagenic and active in inducing InDels in two target genes; however, no large chromosomal fragment deletion was detected. Chromosomal fragment deletion has been reported in kiwifruit with 755 and 271 bp deletions using polycistronic tRNA-gRNA (PTG)/Cas9 system, which is 10-fold higher than conventional CRISPR/Cas9 expression cassette (Wang et al., 2018). The PTG/Cas9 system enables a simultaneous expression of multiple gRNAs up to 31 times higher than monocistronic cassette and improves mutagenesis of multiple chromosomal fragment deletions in rice protoplast (Xie et al., 2015). Moreover, large chromosomal segments of more than 100 kb deletions between two genomic loci were achieved in rice protoplasts using the multiplex gRNA expression system (Zhou et al., 2014). Chromosomal fragment deletion is a potential strategy for chromosomal engineering to remove undesired loci with detrimental traits in crops and could be achieved by multiplex gRNA expression or PTG/Cas9 systems using gRNAs with similar efficiency at both target sites.

Our study demonstrated that the CRISPR/Cas9 genome editing system is effective and efficient in editing oil palm genes. In this study, the established *in vitro* electro-transfection assay provides a rapid assessment and evaluation of gRNA efficiency in oil palm protoplast to reduce the time and cost for transformation and regeneration in oil palm using inefficient gRNAs. An efficient transformation system in oil palm or methods to generate non-transgenic gene-edited mutants are highly desired to enhance the occurrence of targeted mutagenesis with the CRISPR/Cas9 system. This enabling platform for genome editing may accelerate the exploration of gene function for trait improvement in oil palm, in particular, the agronomic traits that address challenges in oil palm cultivation including basal stem rot or *Ganoderma* disease, abiotic stress tolerance to reduce losses due to climate change, and ease of harvesting traits such as dwarf, long stalk, and virescens. In addition, our study offers new insights to other related palm species including date palm and coconut that shared similar limitations in the DNA delivery method, low efficacy of genetic transformation, and inefficient tissue culture propagation and regeneration processes. The availability of full genome sequence opened a new opportunity for genetic improvement in date palm and coconut through the CRISPR/Cas9 genome editing approach. This approach will be helpful to develop date palm and coconut resistance against biotic and abiotic stresses.

## Data Availability Statement

The datasets presented in this study can be found in online repositories. The names of the repository/repositories and accession number(s) can be found in the article/Supplementary Material.

## Author Contributions

W-CY, DRA, and KH conceived the experiments. W-CY constructed the vectors, performed oil palm protoplast experiments, analyzed the data, and wrote the manuscript. NCMK, NJ, and MRM conducted the oil palm transformation and tissue culture and regenerated transgenic oil palm. All authors contributed to the article and approved the submitted version.

## Conflict of Interest

All authors were employed by the company Sime Darby Plantation Berhad.

## Publisher’s Note

All claims expressed in this article are solely those of the authors and do not necessarily represent those of their affiliated organizations, or those of the publisher, the editors and the reviewers. Any product that may be evaluated in this article, or claim that may be made by its manufacturer, is not guaranteed or endorsed by the publisher.

## Footnotes

1
https://benchling.com/

2
https://cctop.cos.uni-heidelberg.de:8043/

3
https://tide.nki.nl/
