# Supplementary material for: An Efficient Clustered Regularly Interspaced Short Palindromic Repeat (CRISPR)/CRISPR-Associated Protein 9 Mutagenesis System for Oil Palm (Elaeis guineensis)
Source: Front Plant Sci. 2021 Nov 22;12:773656. doi: 10.3389/fpls.2021.773656 (PMC8647858; doi:10.3389/fpls.2021.773656)
Supplement: Supplementary file 1 [file Data_Sheet_1.pdf]

Supplementary Material

Data Sheet S1. Sequence of codon optimized *SpCas9* gene

```
>Codon optimized SpCas9
ATGGATAAGAAGTACTCAATCGGCCTCGATATCGGTACAAACAGCGTTGGCTGGGCCGTTATCACCGACGAATATAAGGTTCCGAGCAAGAAGT
TCAAGGTTCTGGGAAACACCGACAGGCACAGCATCAAGAAGAATCTTATAGGGGCTCTCCTTTTCGATAGTGGAGAGACTGCTGAGGCGACCCG
TCTCAAGAGGACCGCTCGTAGGAGGTACACCCGTCGGAAGAATCGTATCTGCTACCTCCAGGAGATCTTCTCAAATGAGATGGCCAAGGTAGAT
GACAGCTTCTTCCACCGACTTGAAGAGTCCTTCCTCGTGGAAGAAGACAAGAAGCATGAGCGTCACCCTATCTTCGGAAACATAGTAGATGAGG
TTGCTTACCATGAGAAGTACCCAACTATCTACCACCTTCGAAAGAAGTTGGTTGATTCCACCGACAAGGCGGATCTCCGCCTTATCTACTTGGC
CTTAGCTCATATGATCAAGTTCCGTGGCCACTTCTTGATCGAGGGCGATTTAAACCCAGATAATAGCGATGTTGATAAGCTATTCATCCAGCTC
GTGCAGACCTACAATCAGTTATTCGAGGAGAACCCTATCAACGCAAGCGGAGTCGATGCAAAGGCGATCCTTTCTGCCCGACTCAGCAAGTCCA
GACGACTCGAGAACCTCATCGCTCAGCTCCCAGGTGAGAAGAAGAACGGCTTATTCGGAAATCTCATCGCACTCTCATTGGGACTCACCCCTAA
CTTCAAGTCAAATTTCGACTTGGCCGAGGATGCTAAGTTACAGCTCTCCAAGGATACCTACGATGACGATCTCGACAACCTCTTGGCGCAGATC
GGTGACCAGTACGCTGATTTGTTCCTCGCCGCTAAGAATCTTTCAGATGCTATCCTCCTTTCCGACATCCTCAGGGTCAACACCGAGATCACCA
AGGCCCCACTCAGCGCTTCCATGATCAAGCGCTACGATGAGCATCACCAGGACTTGACCCTCCTTAAGGCATTAGTTCGACAGCAGCTCCCTGA
GAAGTACAAGGAGATCTTCTTCGACCAGTCAAAGAACGGGTACGCAGGTTACATCGATGGGGGAGCTAGCCAGGAGGAGTTCTACAAGTTCATC
AAGCCAATCTTAGAGAAGATGGATGGAACCGAGGAGTTATTGGTGAAGCTCAATCGTGAGGATCTCCTTCGCAAGCAGAGGACCTTCGACAACG
GCTCTATCCCACATCAGATCCACTTGGGAGAGCTTCATGCCATCTTGAGGAGGCAGGAGGATTTCTACCCTTTCTTAAAGGACAACCGTGAGAA
GATCGAGAAGATCTTGACCTTCCGAATCCCATACTACGTCGGGCCTCTCGCACGTGGCAATAGCCGTTTCGCATGGATGACCCGGAAGTCCGAG
GAGACTATCACCCCATGGAACTTCGAGGAGGTGGTCGACAAGGGTGCTTCAGCACAGTCCTTCATCGAGCGCATGACCAATTTCGATAAGAACC
TTCCAAATGAGAAGGTGCTCCCTAAGCACAGTTTGCTTTACGAGTACTTCACCGTCTACAACGAGTTGACCAAGGTGAAGTACGTCACCGAGGG
GATGCGAAAGCCTGCCTTCCTCTCAGGAGAGCAGAAGAAGGCTATCGTGGATTTACTTTTCAAGACCAATCGAAAGGTCACCGTTAAGCAGTTA
AAGGAGGATTACTTCAAGAAGATCGAGTGCTTCGACAGCGTGGAGATCTCCGGCGTCGAGGATAGATTCAACGCTTCCCTCGGAACCTACCATG
ATTTGCTAAAGATCATTAAGGACAAGGATTTCCTTGATAACGAGGAGAATGAGGACATCTTAGAGGATATCGTGTTAACCTTGACCTTATTCGA
GGACAGGGAGATGATCGAGGAGAGGCTTAAGACCTACGCTCACCTCTTCGACGATAAGGTCATGAAGCAGCTTAAACGTCGCCGTTATACTGGT
TGGGGACGTTTGTCTCGAAAGTTGATCAATGGCATCAGGGATAAGCAGTCTGGCAAGACCATCTTAGATTTCTTGAAGTCCGACGGCTTCGCTA
ACCGCAATTTCATGCAGCTGATCCATGACGATAGTTTGACATTCAAGGAGGACATCCAGAAGGCACAGGTGTCTGGACAGGGTGATAGTCTTCA
TGAGCACATCGCAAACTTAGCCGGGAGCCCAGCCATCAAGAAGGGTATCTTACAGACCGTGAAGGTTGTGGATGAGTTGGTGAAGGTCATGGGC
CGGCACAAGCCTGAGAATATCGTCATCGAGATGGCCCGTGAGAATCAGACCACCCAGAAGGGACAGAAGAACTCCCGAGAGCGTATGAAGCGAA
TCGAGGAGGGCATCAAGGAGTTAGGAAGCCAGATCCTCAAGGAGCATCCTGTTGAGAACACCCAGTTGCAAAATGAAAAGCTCTACCTTTACTA
CCTCCAGAATGGAAGAGACATGTACGTGGATCAGGAGTTAGACATCAACCGTTTAAGTGACTACGATGTCGATCACATCGTGCCACAGAGCTTC
CTTAAGGACGATTCCATCGATAATAAGGTCTTAACCCGTTCTGATAAGAACCGTGGAAAGTCGGACAATGTTCCAAGTGAGGAGGTCGTTAAGA
AGATGAAGAACTACTGGAGACAGCTCCTTAATGCCAAGTTAATCACCCAGCGTAAGTTCGACAACTTAACGAAGGCTGAGCGTGGCGGCTTGAG
TGAGCTTGATAAGGCAGGCTTCATCAAGCGCCAGTTGGTCGAGACTCGCCAGATCACCAAGCATGTGGCTCAGATCTTGGATAGCCGCATGAAC
ACCAAGTACGACGAAAATGATAAGCTTATCCGAGAGGTTAAGGTGATTACCTTAAAGTCTAAGTTAGTCTCCGATTTCCGAAAGGACTTCCAGT
TCTACAAAGTTCGTGAGATTAACAATTACCATCATGCTCACGATGCATACCTCAACGCAGTGGTCGGGACTGCTTTGATTAAGAAGTACCCAAA
GCTCGAGTCGGAGTTCGTGTACGGTGATTACAAGGTTTACGACGTGCGTAAGATGATCGCTAAGTCCGAGCAGGAGATCGGAAAGGCAACCGCT
AAGTACTTCTTCTACTCTAACATCATGAATTTCTTCAAGACCGAGATCACCCTTGCAAATGGAGAGATCCGCAAGCGCCCTCTCATCGAGACTA
ACGGCGAGACTGGAGAAATTGTCTGGGATAAGGGGCGAGATTTCGCCACAGTGCGCAAAGTATTGTCCATGCCACAAGTGAATATCGTCAAGAA
GACCGAGGTGCAGACCGGCGGATTCAGCAAGGAGTCCATCCTTCCTAAGAGGAACTCGGACAAGCTCATCGCCCGTAAGAAGGATTGGGACCCA
AAGAAGTACGGGGGTTTCGATAGCCCTACGGTAGCTTACTCCGTTCTTGTTGTGGCAAAGGTTGAGAAGGGGAAGTCGAAGAAGTTAAAGTCCG
TTAAGGAGTTACTTGGTATCACCATCATGGAGAGAAGCTCCTTCGAGAAGAATCCGATCGACTTCTTAGAGGCTAAGGGATATAAGGAGGTGAA
GAAGGATTTAATCATCAAGCTCCCTAAGTACAGCCTCTTCGAGTTAGAGAACGGACGTAAGCGGATGCTTGCAAGTGCCGGGGAGTTACAGAAG
GGAAATGAGCTCGCCCTGCCAAGCAAGTACGTCAACTTCTTATACTTAGCTAGCCATTACGAGAAGTTGAAGGGCAGTCCTGAGGACAACGAGC
AGAAGCAGTTGTTCGTGGAGCAGCATAAGCACTACCTCGATGAGATCATCGAGCAGATCAGCGAGTTCTCCAAGAGGGTGATCTTAGCAGATGC
CAATTTAGACAAGGTCCTCAGTGCATACAACAAGCATAGGGACAAACCAATCCGTGAGCAGGCAGAGAATATCATCCATTTATTCACGTTGACG
AATCTCGGAGCTCCCGCTGCCTTCAAGTACTTCGATACAACAATTGATCGTAAACGATATACGTCTACAAAGGAGGTGTTAGATGCCACTCTCA
TCCATCAGTCCATCACTGGACTGTACGAGACACGCATTGACTTGAGTCAGCTAGGAGGTGACTAG
```
