# Supplementary material for: An Efficient Clustered Regularly Interspaced Short Palindromic Repeat (CRISPR)/CRISPR-Associated Protein 9 Mutagenesis System for Oil Palm (Elaeis guineensis)
Source: Front Plant Sci. 2021 Nov 22;12:773656. doi: 10.3389/fpls.2021.773656 (PMC8647858; doi:10.3389/fpls.2021.773656)
Supplement: Supplementary file 2 [file Data_Sheet_2.pdf]

SUPPLEMENTARY MATERIAL

Table S1. sgRNAs for *EgPDS* and *EgBRI1* genes

Gene	sgRNA	Guide sequence	PAM	Position	GC%
EgPDS	gPDS1	AATTGTGCACGGCCGAGTAA	AGG	321	50
	gPDS2	TACTCTCTTGGCATAGATTC	TGG	102	40
	gPDS3	TTGTCTGCATGGACTATCCA	AGG	238	45
	gPDS4	GCAGCTTGGAAGGATGAAGA	TGG	444	50
	gPDS5	GCTTGCCCCCCAACCATGGC	AGG	720	70
EgBRI1	gBRI1	TACAGTCCCCGAGCTCCGGC	GGG	1857	70
	gBRI2	CTGGGCCACAATATGCTCTC	CGG	2026	55
	gBRI3	GGATCGGCCGGCTCGGCAAT	AGG	1406	70
	gBRI4	ACCTCGCAGGTAATGGCCTC	GGG	409	60
	gBRI5	TTCGAGACTCTAGTCTCTTC	GGG	886	45

Table S2. Primers used for molecular confirmation of gene editing event

**Gene**	**sgRNA**	**Forward Primer Sequence**	**Reverse Primer Sequence**
EgPDS	gPDS1	GACTATCCAAGGCCTGAACT	GCAAGATATTTTGCTGTAGA
	gPDS2	ATACCATTGGATTTATCTCC	GGCTTGATCTTGTTCCTTTG
	gPDS3	TAGCGAGTTTATGGGTTGCC	GACAGTATTCTCAAGTTCAG
	gPDS4	ACCACCTTCTCCAAATGGCT	TGATGATCGGCACATGAGGA
	gPDS5	AGGCATCATCCTGCTGACAT	TGGCCAACCATCATGTTCTCA
EgBRI1	gBRI1	CTCGATCTCTCTTACAACCA	TCCAAGACACCAACATAGTG
	gBRI2	CGAAGGTCCGATCCCAGCTT	TGCCATTAGCACTCTGTCCA
	gBRI3	CCTCAGCAACTGCACCGATT	GGAGAAAGAATTGTTCCCGA
	gBRI4	GCCAACCTCACCGGAAATAT	AGTTGCCGGAGAGATTGAGG
	gBRI5	CTACCTGAACCTCTCTGCCA	AGGGGCCCAGAGAAGTTGTT

Table S3. Primers for PCR confirmation of transgene

**Gene**	**Forward Primer Sequence**	**Reverse Primer Sequence**
Cas9	AGAATCTTAGCGATGCTATCCT	ACCTGCTATTACCCCTTGCG
OsU3	AGGCGTCTTCTACTGGTGCT	CGAACAGGCTTATGTCCACT
