# Supplementary material for: An Efficient Clustered Regularly Interspaced Short Palindromic Repeat (CRISPR)/CRISPR-Associated Protein 9 Mutagenesis System for Oil Palm (Elaeis guineensis)
Source: Front Plant Sci. 2021 Nov 22;12:773656. doi: 10.3389/fpls.2021.773656 (PMC8647858; doi:10.3389/fpls.2021.773656)
Supplement: Supplementary file 3 [file Data_Sheet_3.PDF]

Supplementary Material

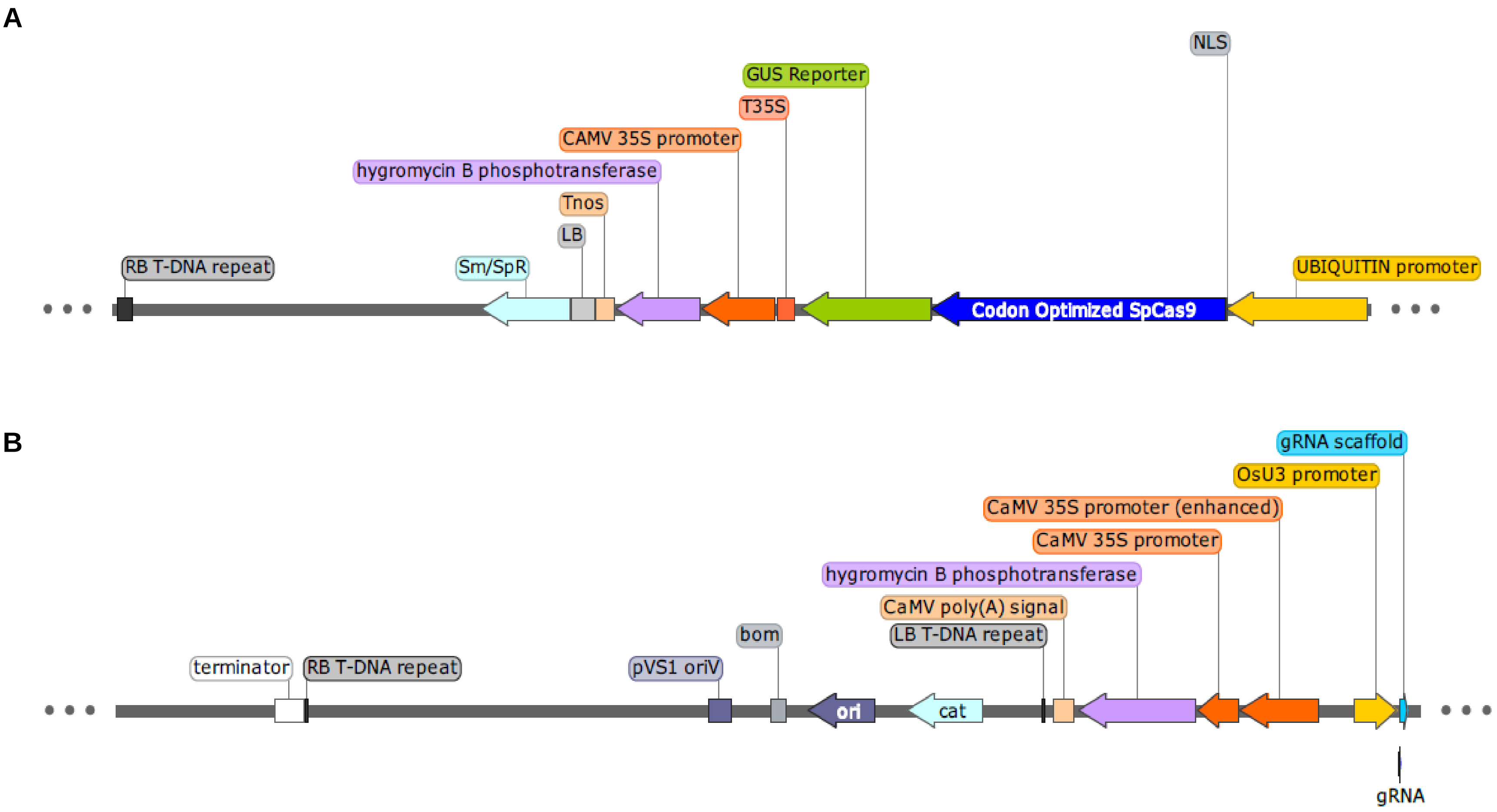

Figure S1. Scheme of binary plasmids for CRISPR/Cas9 gene editing. **(A)** pUbil-Cas9 plasmid containing codon optimized *SpCas9* fused to nuclear localization signal SV40 (NLS) and *β-glucuronidase* (*GUS*) reporter gene and expression driven by *ubiquitin* promoter. **(B)** Expression of single guide RNA (gRNA) for *EgPDS* and *EgBRI1* target sites driven by *OsU3* promoter from *Oryza sativa* and fused to the gRNA scaffold cassette. Both plasmids carry the *hygromycin B phosphotransferase* gene as selection marker under the control of constitutive cauliflower mosaic virus 35S (CaMV 35S) promoter. LB, left border; RB, right border

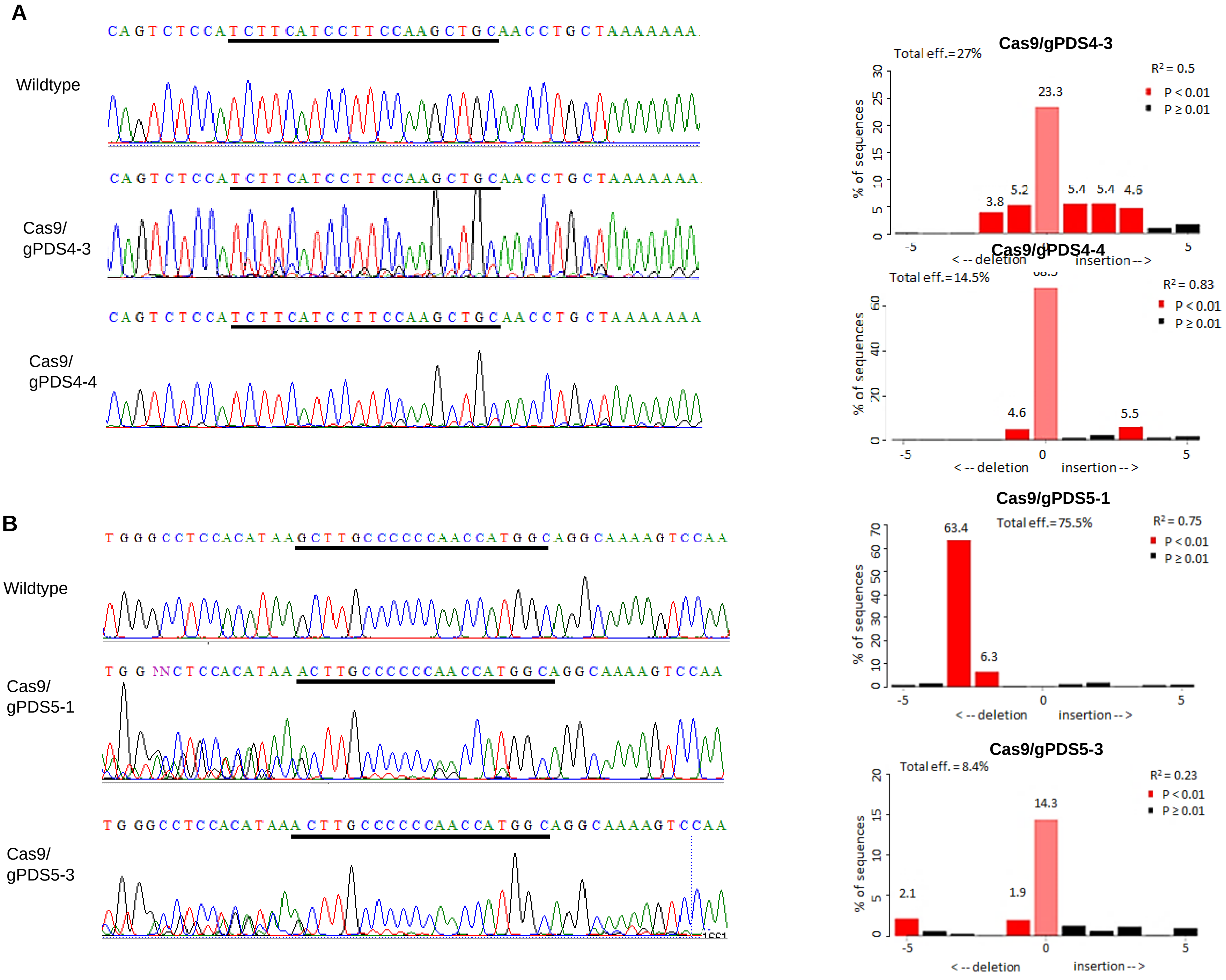

Figure S2. *In silico* sequence analysis of CRISPR/Cas9-mediated mutagenesis on *EgPDS* gene. The mutation on *EgPDS* targeted sites were analyzed using Sanger sequencing and the occurrence of insertion and deletion events was qualitatively detected using TIDE algorithm using the electropherogram for **(A)** Cas9/gPDS4 and **(B)** Cas9/gPDS5 mutation regions.
